# Utilizing the Operating Room Black Box to Characterize Intraoperative Delays, Distractions, and Threats in the Gynecology Operating Room: A Pilot Study

**DOI:** 10.7759/cureus.16218

**Published:** 2021-07-06

**Authors:** Alysha Nensi, Vanessa Palter, Cheyanne Reed, Pansy Schulthess, Mary Mcloone, Teodor Grantcharov, Eliane M Shore

**Affiliations:** 1 Department of Obstetrics and Gynecology, St. Michael’s Hospital, Toronto, CAN; 2 Department of Surgery, St. Michael’s Hospital, Toronto, CAN; 3 Obstetrics and Gynecology, University of Toronto, Toronto, CAN; 4 Department of Nursing, St. Michael’s Hospital, Toronto, CAN; 5 Department of Anesthesia, St. Michael’s Hospital, Toronto, CAN

**Keywords:** quality improvement, patient safety, efficiency, gynecology, hysterectomy

## Abstract

Introduction

Operating Room Black Box (ORBB) technology can be used to capture information during surgery for analysis and potential identification of root causes that jeopardize safety and efficiency. In this study, our objective was to identify and characterize procedural steps, intraoperative distractions, errors, and threats, as well as the non-technical skills of the team during a common minimally invasive gynecologic procedure.

Methodology

This was a cross-sectional pilot study of 25 patients undergoing total laparoscopic hysterectomy between May 2019 and February 2020 at a Canadian tertiary care academic hospital. Video, audio, and patient physiologic data from all procedures were obtained through a multichannel synchronized recording device (ORBB). Trained analysts reviewed and coded the recordings.

Results

The median total case time was 165 minutes (interquartile range [IQR]: 160-178 minutes) with the shortest step being cystoscopy and the longest being vaginal cuff closure. Time pressure and device absence or malfunction occurred in 48% of the cases, and a median of 262 (IQR: 228-304) auditory distractions were noted per case. There was a median of 3 (IQR: 2-4) safety threats identified per case and at least one error was identified in 11/25 cases (44%). Only two adverse events were noted among all 25 cases. Observed non-technical skills were mainly positive, and observations were the highest for situational awareness and leadership among the surgical team and communication and teamwork among the nursing/scrub technician and anesthesia teams.

Conclusions

This study is a novel application of the ORBB in the gynecology operating room to capture information regarding procedure times, intraoperative distractions, errors, and non-technical skills of the team. Frequent intraoperative cognitive and auditory distractions were noted. Although adverse events were rare, safety threats were identified. Ongoing and future research from our group will aim to identify key areas for organizational, technological, and team improvement to minimize inefficiencies and optimize patient safety in the operating room.

## Introduction

Major surgical complications are common and occur in 3-17% of the over 200 million yearly worldwide operations, depending on surgical specialty and case acuity [1,2]. A medical error is defined by the Institute of Medicine as “the failure to complete a planned action as intended or the use of a wrong plan to achieve an aim,” while adverse events are “an injury caused by medical management rather than by the underlying disease or condition of the patient” [3]. Not all errors result in adverse events, and not all adverse events or complications are due to an error; however, approximately half of all surgical complications are due to human factors or systems errors, making them potentially avoidable [4-6].

Many factors contribute to surgical adverse events in the operating room. To date, however, our understanding of the etiology of these events is limited by the inability to accurately capture data from the intraoperative environment. The majority of information describing adverse events in the operating room is retrospective and must be gleaned from operative reports, incident reports, or notes from morbidity and mortality rounds. These methods are prone to bias and are often overly generalized or incomplete [7,8]. In addition, errors and near misses are often not documented at all, mostly because they are rectified before any harm to the patient.

Patient safety and quality of surgical care can also be impacted by inefficiency in the operating room. Time delays in the operating room can result in increased surgical wait times, a higher number of canceled cases, increased healthcare expenditure, and wasted resources [9].

Improvements in surgical efficiency and patient safety require the ability to perform an objective review and analysis of intraoperative events. Understanding the distractions and events which occur in the operating room, the frequency and trends in which they occur, and their root causes may help prevent delays or errors through anticipation and mitigation. For this purpose, the Operating Room Black Box (ORBB) has been developed by Surgical Safety Technologies Inc. (Toronto, ON, Canada). This technology facilitates the synchronous capture of audio, video, and patient physiologic data in the operating room. This project is a pilot study that aimed to utilize ORBB technology to determine total case times, identify intraoperative distractions, threats, and errors, as well as describe observed non-technical skills of the team during a common minimally invasive gynecologic procedure.

## Materials and methods

Study design

This was a cross-sectional study conducted from May 2019 to February 2020, during which we examined data from 25 total laparoscopic hysterectomies (TLH) performed at a Canadian tertiary care academic hospital. All procedures were performed by generalist obstetrician-gynecologists with clinical fellows or residents acting as the first assist. The objectives of our study were to identify and characterize procedural steps, intraoperative delays, distractions, and threats in the operating room, as well as to describe the observed non-technical skills of the surgical, anesthesia, and nursing/scrub technician teams using the ORBB.

Study subjects

Patients undergoing TLH and bilateral salpingectomy with or without oophorectomy were included. Procedures were excluded if data collection was in any part incomplete or consent was declined or withdrawn, either by the patient or the operating room staff. Minors, pregnant individuals, and patients with mental disabilities or impairments were not eligible for the study. Cases with severe endometriosis, history of significant adhesive disease, uteri greater or equal to 17 weeks size, body mass index (BMI) over 40, or previous midline laparotomies were also excluded to minimize patient-related variability in the surgical procedures.

Data collection

Patient demographic characteristics including patient age, American Society of Anesthesiologist class score, BMI, procedure type, procedure indication, and previous surgical history were obtained from clinic notes and preoperative and intraoperative anesthesia notes through the electronic medical records.

The ORBB captured data from a number of sources including audio feeds from ceiling-mounted microphones, video feeds from the laparoscopic camera as well as four wall-mounted room cameras, and patient physiologic data from the anesthesia monitor. The data streams were synchronized, encrypted, and stored on a secure server. Recording began when the patient entered the operating room and ended when the patient exited the operating room. The completed recording was assigned a random case number linked to the patient log, which was kept on a secure server for 30 days, after which both the recording and the log were deleted. Two expert analysts, both board-certified gynecologists who received at least three months of training to administer the protocol and had at least two years of experience in analyzing intraoperative data, reviewed the recordings and identified intraoperative factors as per the standardized protocol.

Outcomes

The following intraoperative factors were identified and characterized:

1. Each case was divided into nine procedural steps: access, transection of the adnexa, dissection of the bladder flap, coagulation and section of uterine pedicles, colpotomy, specimen removal, vaginal cuff closure, cystoscopy, and closure. These steps were identified via a multidisciplinary working group at our center consisting of gynecologic surgeons, an anesthetist, an operating room nurse, and ORBB systems analysts.

2. Intraoperative distractions were identified and characterized according to a modified Disruptions in Surgery Index [10] and included door openings, operations updates (internal and external), machine alarms, phones, personal pagers, and overhead paging.

3. Intraoperative adverse events errors and threats were characterized according to the Intraoperative Adverse Event Severity Classification Scheme published by Kaafarani et al. [11].

4. Non-technical skills were assessed using the Non-technical Skills for Surgeons (NOTSS), Anaesthetists’ Non-technical Skills (ANTS), and Scrub Practitioners’ List of Intraoperative Non-technical Skills (SPLINTS) frameworks for the surgical, anesthesia, and nursing/scrub technician teams, respectively. The scales were used to identify non-technical skills and categorize them as either a positive (+) or a negative (-) interaction. All three rating scales have previously been used in surgical teams and have been shown to have construct validity and good interrater reliability [12-14]. Non-technical skills include teamwork, communication, leadership, situational awareness, decision-making, and task management.

Statistical analysis

All data analyses were conducted using SPSS version 25 (IBM Corp., Armonk, NY). Continuous data are presented as means ± standard deviations and medians (interquartile range [IQR]), and categorical data are presented as frequencies (percentage).

Ethical approval

This study was approved by the Unity Health Research Ethics Board (REB#17-029).

## Results

During the study period, there were 37 elective TLHs that met the inclusion criteria, of which 25 were included in the final analysis. Of the 12 cases that were not included in the final analysis, three were due to operating room staff not consenting to the study, seven were due to the procedure being moved to a different operating room that did not have the ORBB installed, one was due to the patient not consenting, and one was due to a malfunction in the ORBB control panel.

Patient and procedure characteristics are summarized in Table 1. The most common procedure was TLH with bilateral salpingectomy (76%) and the most common surgical indications were leiomyoma (48%), fibroids (36%), and malignancy (36%).

**Table 1:** Patient and procedure characteristics. BMI: body mass index; ASA: American Society of Anesthesiologists; TLH/BS: total laparoscopic hysterectomy, bilateral salpingectomy; TLH/BSO: total laparoscopic hysterectomy, bilateral salpingo-oophorectomy

Patient characteristics	n = 25
Age, years, mean (SD)	48 ± 11
BMI, kg/m^2^, mean (SD)	28 ± 6
ASA class, n (%)	
1	3 (12)
2	12 (48)
3	10 (40)
4	0 (0)
Procedure type, n (%)	
TLH/BS	19 (76)
TLH/BSO	6 (24)
Procedure indication, n (%)	
Adenomyosis	2 (8)
Leiomyoma	12 (48)
Malignancy	9 (36)
Endometrial	1 (4)
Transition-related surgery	1 (4)
Previous abdominal surgery, n (%)	
Yes	11 (44)
No	14 (56)

Procedure duration

**Figure 1:**
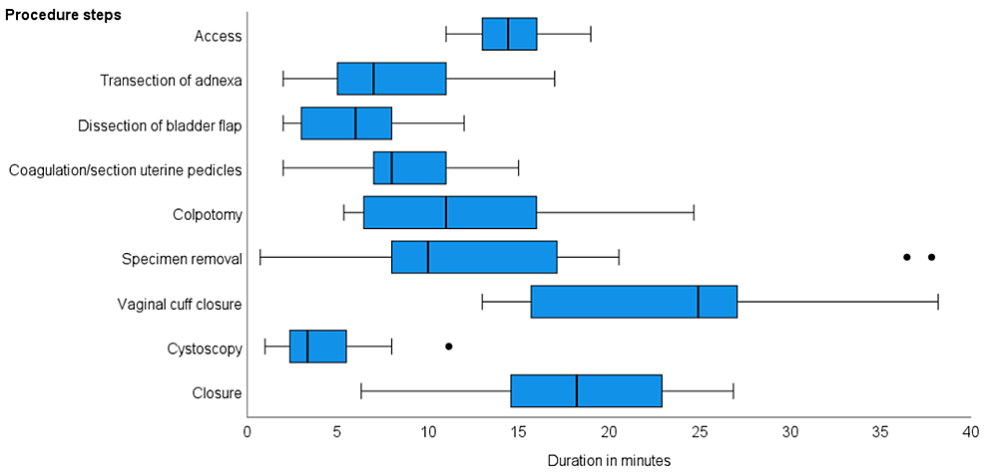
Box-and-whisker plot of the duration of the procedure steps of total laparoscopic hysterectomy. The median value is presented as a horizontal line while the boxes represent the interquartile range (Q1-Q3). The whiskers depicted as vertical lines represent 1.5 times the interquartile range and the circles represent outliers.

The median procedure duration was 165 minutes (IQR: 160-178 minutes). The longest procedure was 250 minutes and the shortest was 148 minutes. The duration of the various procedure steps is displayed in Figure 1. The shortest procedure step was cystoscopy, with a median time of 3.3 minutes (IQR: 2.4-5.5 minutes) and the longest step was vaginal cuff closure, with a median time of 25.0 minutes (IQR: 15.7-27.1 minutes). Specimen removal lasted a mean duration of 10.0 minutes (IQR: 8.0-17.1 minutes) with two notable outliers of 36.5 and 37.8 minutes. Both of these cases required abdominal morcellation of a large uterine specimen through a mini-laparotomy.

Distractions

Intraoperative distractions, including both cognitive and auditory distractions, are presented in Table 2. There was time pressure observed in 48% of cases, and non-case-related conversation occurred in the majority (64%) of cases. There were absent or malfunctioning devices in 48% of the cases. There was, on average, a noise-related auditory distraction once every minute. The operating room door opened a median of 89 times per case. There was a median of 158 (IQR: 145-179) machine alarms per case, 10 (IQR: 4-13) incoming phone calls to the operating room phone, and 3 (IQR: 1-4) pages per case.

**Table 2:** Characteristics of intraoperative distractions. *Time pressure refers to any inquiry received (internally or externally) regarding the estimated time of case completion. IQR: interquartile range

Cognitive distractions	
Case with any time pressure*, n (%)	12 (48)
Case with non-case-related conversation, n (%)	16 (64)
Case with any absent or malfunctioning device, n (%)	12 (48)
Auditory distractions	
Door openings per case, count, median (IQR)	89 (77-104)
Machine alarms per case, count, median (IQR)	158 (145-179)
Incoming OR phone calls per case, count, median (IQR)	10 (4-13)
Noise from personal phone or pager per case, count, median (IQR)	3 (1-4)
Noise from overhead paging system per case, count, median (IQR)	2.5 (1-4)

Intraoperative adverse events, errors, and threats

Among all 25 cases, a total of 23 errors were noted. At least one error was identified in 11 (44%) cases. The most common error was inadequate cauterization of the uterine arteries which resulted in unexpected bleeding during either uterine artery transection (4/25 [16%] cases) and/or colpotomy (9/25 [36%] cases). Two intraoperative adverse events were noted among the 25 cases, both instances were injuries to the bladder with a sharp instrument secondary to inadequate visualization (class I). Neither injury resulted in cystotomy or required any repair of bladder serosa. There was a median of 3 (IQR: 2-4) threats identified per case, with inadequate visualization noted 34 times in 18 different cases, loss of pneumoperitoneum identified nine times in nine different cases, tool failure identified 18 times in 12 cases, and technical threats (i.e., risk of injury or adverse event due to actions of the surgeon) identified 14 times in 12 cases.

Observed non-technical skills

The observed non-technical skills for surgeons, nurses, and anesthetists are summarized in Table 3 using the NOTSS, SPLINTS, and ANTS frameworks, respectively. The vast majority of observations for all three groups were positive. Positive observations were highest in situational awareness and leadership among the surgical team and communication and teamwork among the nursing/scrub technician and anesthesia teams. The most common negative observation for the surgical team was in setting/providing and maintaining standards, and all 13 negative observations were related to a lack of maintaining sterile technique. There were no negative observations noted for the nursing/scrub technician or anesthesia teams.

**Table 3:** Summary of observed non-technical skills. NOTSS: Non-technical Skills for Surgeons; SPLINTS: Scrub Practitioners’ List of Intraoperative Non-technical Skills; ANTS: Anaesthetists’ Non-technical Skills

	NOTSS		SPLINTS		ANTS	
Type of observation	+	-	+	-	+	-
Situational awareness	103	8	16	0	12	0
Decision-making	38	0	-	-	2	0
Communication and teamwork	99	1	23	0	24	0
Task management	-	-	8	0	4	0
Leadership	103	13	-	-	-	-

## Discussion

The utilization of the ORBB platform allowed us to capture objective intraoperative data during this cross-sectional study of patients undergoing TLH. We were able to determine which steps during the procedure were the longest and the shortest and which had the highest degree of variability. This information is important in helping the surgical team determine the expected flow of the case, anticipate completion times for each surgical step, and estimate finishing time for the entire procedure. These details also allow for the identification of steps that have the potential to cause the most time delay, for example, in our study, it was noted that while specimen extraction took a median time of 10 minutes, there were two cases in which specimen extraction took over 35 minutes. This represents an area for improvement and can flag the surgical team to review this step during these two cases.

Our study demonstrated that cognitive and auditory distractions were extremely common in the operating room, with the door opening once every 1.8 minutes and a noise-related auditory distraction occurring on average once every 60 seconds. Distractions have been shown to have a negative effect on surgeon precision and problem-solving, as well as increasing stress levels among surgical trainees [15-17]. In addition, door opening and frequent turnover of personnel may also increase the risk of surgical site infections [18,19]. While occasional door openings and certain machine alarms cannot be eliminated, there were also frequent unnecessary distractions such as non-case-related conversations which occurred in 64% of cases, and absent or malfunctioning devices which occurred in 48% of cases. These results parallel those reported in the general surgery operating room by Jung et al. who noted auditory distractions once every 40 seconds and device-related interruptions in 33% of cases [20]. A future study by our group will aim to address the impact of various distractions on team performance as well as patient outcomes.

The ORBB was also able to capture occurrences of intraoperative adverse events, errors, and threats. While the overall rate of errors was low, at least one error was identified in 44% of cases. The vast majority of errors were related to unexpected bleeding, either during the ligation of the uterine arteries or during the colpotomy. All of these instances were rectified with no resultant adverse events; however, these types of errors are often not captured in the operative record or traditional outcome measures but represent an area in which technical skills can be improved. In addition, tool failure, which was a common threat noted, may also play a role in inadequate vessel ligation, highlighting another area of potential improvement. Other latent safety threats noted during the procedure included inadequate visualization, loss of pneumoperitoneum, and technical threats. The two adverse events that occurred were both class I injuries to the bladder which did not require organ removal or a change in the initially planned procedure. Targeted interventions that can address these errors and latent safety threats, such as appropriate equipment selection and education for trainees and practicing physicians around these potential safety threats and ways to mitigate them may help ensure that cumulative threats do not eventually result in errors or adverse events.

Finally, our study was able to capture the non-technical skills of the team in an objective and unobtrusive manner. Previous criticism of studies that measure and categorize team dynamics suggests that the most common methods used, such as direct observation by intraoperative research personnel, may not represent the true team performance, due to the intrusive nature and the inability to capture information from a large number of activities occurring simultaneously [21]. Analysis using the ORBB eliminated these barriers and allowed for a comprehensive analysis of team non-technical skills. The vast majority of the observations were positive, but specific areas for improvement, such as infection control practices, were also noted and can be used by teams to optimize their performance.

Limitations of our study included the low number of cases that were captured, which may have been due to issues with obtaining consent, as there were concerns noted by some patients and operating room personnel regarding privacy and medicolegal concerns. Additionally, the pilot project was stopped prematurely secondary to the coronavirus disease 2019 pandemic which limited elective surgeries at our center, access to the operating room containing the ORBB, and the ability for research volunteers to enter the hospital and obtain consent from patients and operating room staff. Nevertheless, this pilot study indicated that our project is feasible, and we are continuing to provide education to patients and surgical team members to address their concerns. Future studies can expand to capturing a larger variety of cases to make our results more generalizable and increase case numbers. The final limitation is due to the Hawthorne effect, which describes a phenomenon of an unintentional change in behavior in response to the presence of an observer. The Hawthorne effect can affect any study that involves observation of the study subjects and can therefore not be eliminated [22]. It is known that the Hawthorne effect typically fades with time, as the subjects get used to the observation, especially if the presence of an observer is not directly visible. We attempted to mitigate the Hawthorne effect in our study by utilizing a long study period and ensuring that the recording devices in the operating room were small and non-obtrusive.

## Conclusions

Our study is a novel application of the ORBB in the gynecology operating room to capture information regarding procedure times, intraoperative distractions, errors and threats, and the non-technical skills of the team. We were able to successfully identify variations in procedural steps and characterize the non-technical skills of the team. Frequent intraoperative cognitive and auditory distractions were noted. Although error rates were low, safety threats were identified which provides areas for opportunity. Ongoing and future research from our group will aim to identify key areas for organizational, technological, and team improvement to ultimately develop and implement standardized and targeted interventions to minimize inefficiencies and optimize patient safety in the operating room.
